# Presumed first episode of nonconvulsive status epilepticus as the cause of postoperative disorder of consciousness following the completion of general anesthesia: A case report

**DOI:** 10.1002/ccr3.7988

**Published:** 2023-09-27

**Authors:** Yumi Tsuzuki, Yusuke Ishida, Mikiko Tomino

**Affiliations:** ^1^ Department of Anesthesiology Tokyo Medical University Tokyo Japan

**Keywords:** disorder of consciousness (DOC), electroencephalogram (EEG), general anesthesia, nonconvulsive status epilepticus (NCSE)

## Abstract

**Key Clinical Message:**

Disorder of consciousness can lead to irreversible sequelae without proper intervention. Consequently, early diagnosis and treatment are of paramount importance in patients with disorder of consciousness.

**Abstract:**

Disorder of consciousness (DOC) has various etiologies. Here, we report a case in which DOC following general anesthesia was suspected as being due to the first episode of nonconvulsive status epilepticus (NCSE). An elderly man in his 80s underwent uneventful tumor resection surgery under general anesthesia for extramammary Paget's disease. After the procedure, he regained consciousness following anesthesia discontinuation and was extubated. Soon after extubation, however, although his respiratory status remained stable, his level of consciousness deteriorated to a Glasgow Coma Scale (GCS) score of E1V1M1. Head computed tomography and magnetic resonance imaging scans indicated no abnormal findings. Subsequently, involuntary movements were noted in his left upper limb. Suspecting an epilepsy episode, diazepam was administered, leading to an improvement in the level of consciousness (GCS: E4V5M6). Based on the improvement in consciousness after diazepam administration, we strongly suspected NCSE.

## Graphical abstract

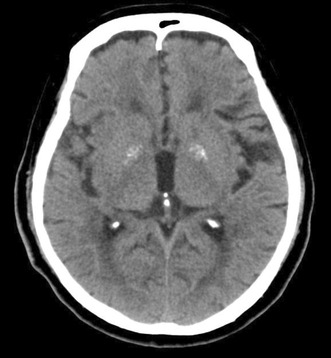

## 1 BACKGROUND

Disorder of consciousness (DOC) is a state of prolonged altered consciousness that can be categorized into coma, vegetative state, or minimally conscious state based on neurobehavioral function.^1^ The causes of DOC are diverse and can include cerebrovascular disorders, cardiac conditions, drug intoxication, hypoglycemia, infections, and psychiatric abnormalities.^2^ DOC can lead to irreversible sequelae if early intervention is not provided. Therefore, early diagnosis and treatment are of paramount importance.^3^ Indeed, diagnosing DOC during the perioperative period can be challenging due to the effects of anesthesia. We encountered a patient who developed impaired consciousness after surgery, following awakening and extubation. Based on his clinical presentation, a first episode of nonconvulsive status epilepticus (NCSE) was considered as the cause of his DOC. Here, we provide a report of this case along with a literature review to support our findings and contribute to the existing knowledge on this topic. The patient's written, informed consent was obtained for publication of this case report.

## 2 CASE PRESENTATION

The patient was an elderly man in his 80s, 148 cm tall, weighing 50.0 kg, who presented to a nearby clinic with discomfort in the inguinal region. Further investigation led to a diagnosis of extramammary Paget's disease, for which surgical excision under general anesthesia was planned. The patient had a medical history of hypertension and asymptomatic cerebral infarction and was on aspirin. He had no significant family history. Electrocardiography revealed complete right bundle branch block, and blood tests did not show any notable abnormalities.

**FIGURE 1:**
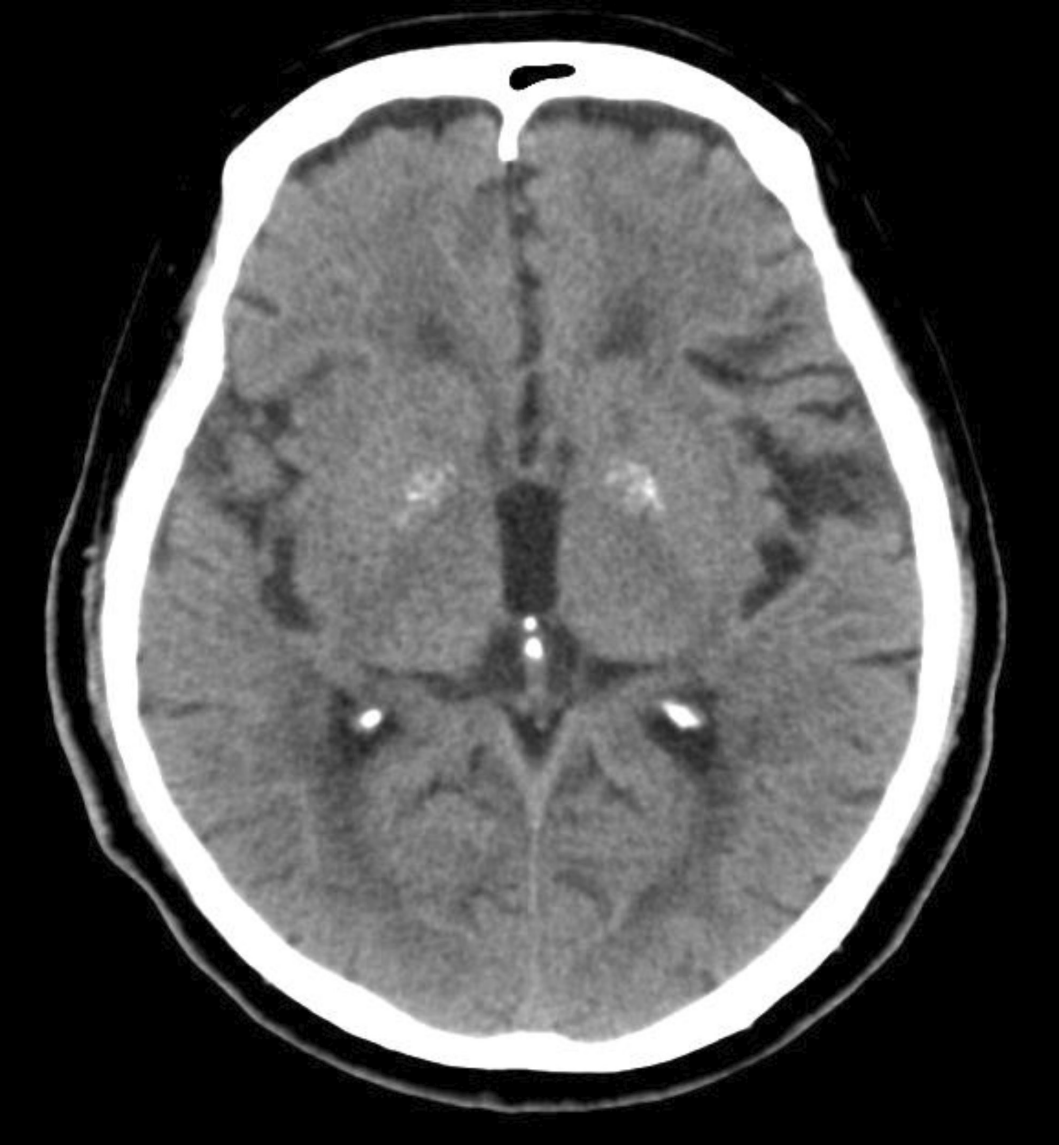
Head computed tomography: There was no evidence of intracranial hemorrhage.

**FIGURE 2:**
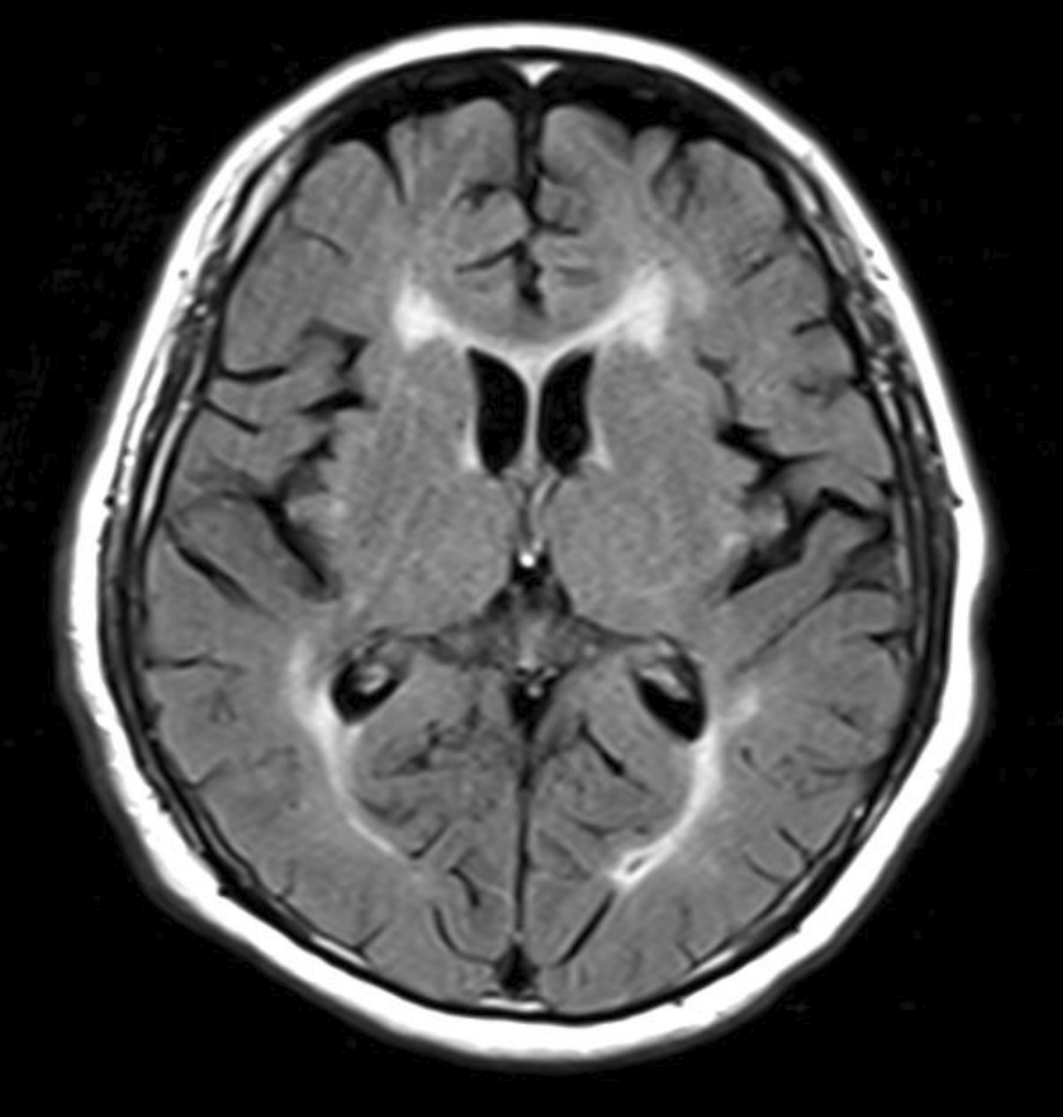
Head magnetic resonance imaging: There were no findings indicating new‐onset infarction.

**FIGURE 3:**
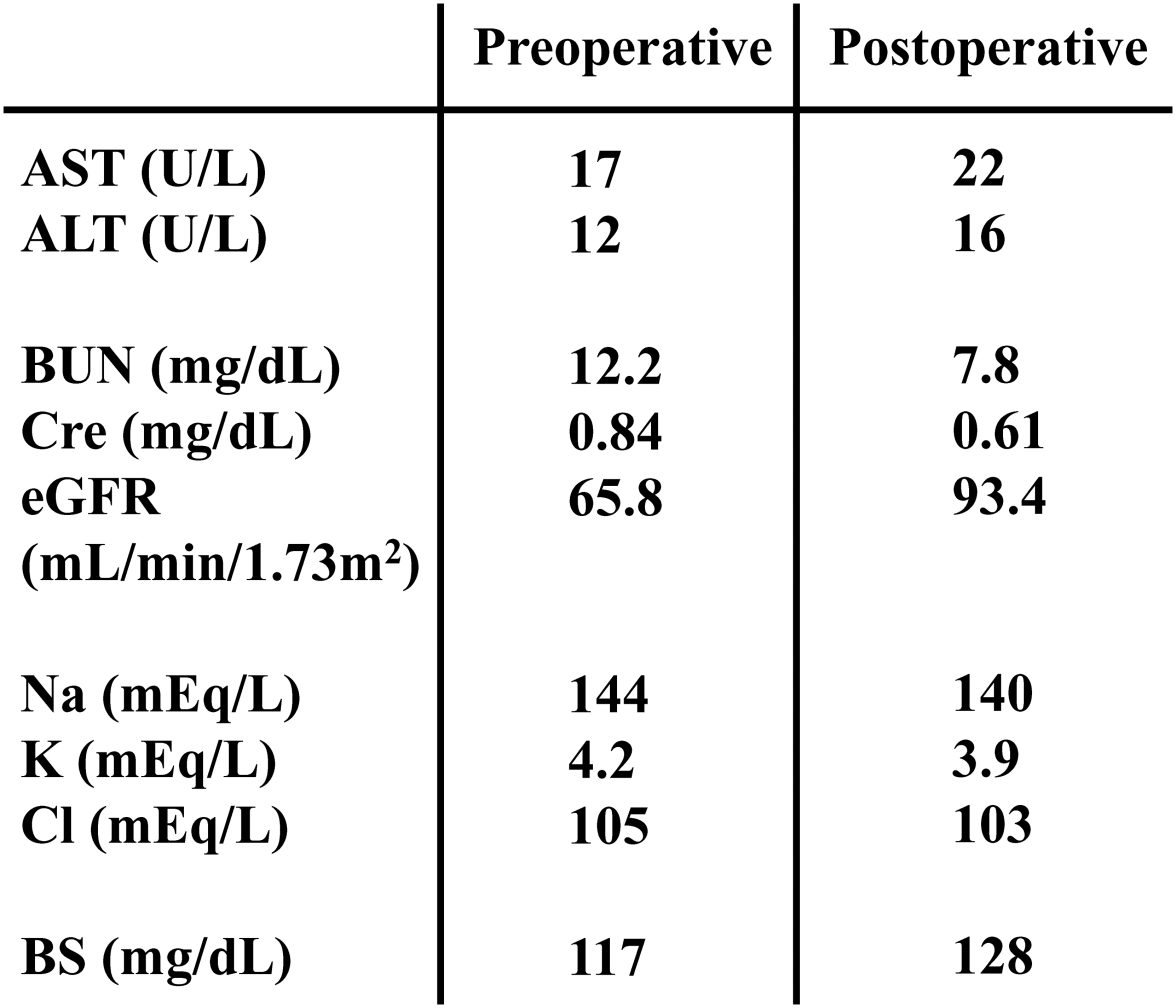
Blood test: There was no change in liver and kidney function before and after surgery.

Anesthesia induction was performed with propofol 60 mg, fentanyl 100 μg, remifentanil 0.1 μg/kg/min, and rocuronium 50 mg. Maintenance of anesthesia was achieved using sevoflurane 1%, remifentanil at a rate of 0.1–0.15 μg/kg/min, and intermittent administration of rocuronium 10 mg. An 8‐mm endotracheal tube was used for intubation. Intraoperatively, the patient's hemodynamics and oxygenation were stable without any significant abnormalities. Brain function monitoring to assess sedation levels was not performed intraoperatively and was not used to assess sedation levels. The surgery lasted for 2 h and 49 min. The surgical procedure was completed uneventfully, following which the administration of anesthesia was discontinued. When the end‐tidal concentration of sevoflurane reached 0.1%–0.2%, the patient was awakened, and eye opening was observed. After confirming the patient's consciousness, sugammadex was administered, and extubation was performed. A few minutes after extubation, the patient's level of consciousness was observed to decrease, subsequently reaching a Glasgow Coma Scale (GCS) score of E1V1M1. His respiratory status remained stable. Considering the possibility of the effect of residual opioids, naloxone was administered, but with no change in the level of consciousness. His body temperature was 37.0°C. Prompt blood gas analysis was performed, which revealed normal pH and electrolyte levels. There were no signs of hypoglycemia (blood sugar 110 mg/dL) and no specific findings to explain the decrease in consciousness level. However, due to the emergence of right conjugate eye deviation, further investigations were conducted to evaluate for an intracranial pathology. Both head computed tomography and magnetic resonance imaging scans were performed, but no abnormal findings, such as new brain infarctions or intracranial hemorrhage, were detected (Figures 1 and 2). Since there were no respiratory or circulatory abnormalities, re‐intubation was not performed. Preoperative systolic blood pressure was around 120 mmHg, and intraoperative systolic blood pressure remained at 100–130 mmHg. The patient was urgently transferred to the intensive care unit (ICU) for close observation. Approximately 1 h after admission to the ICU, the patient developed involuntary movements in the left upper limb, suggestive of a seizure. Based on the assumption of an epileptic seizure, 5 mg of diazepam was administered, which resulted in an improvement in the level of consciousness (GCS E4V5M6). No further obvious neurological abnormalities were observed. In addition, there were no changes in liver and kidney function before and after surgery (Figure 3). Subsequently, levetiracetam administration was initiated from the first day after surgery. Electroencephalography (EEG) performed on the second day after surgery showed minimally significant findings. No seizures occurred thereafter, and the patient was discharged on the 16th day after surgery without any neurological complications. No other complications were observed during his hospitalization.

## 3 DISCUSSION

In this case, the patient developed unexplained postoperative DOC, which was suspected to be an epileptic seizure based on the diagnostic and therapeutic intervention. The causes of DOC can include cerebrovascular disorders, cardiac conditions, drug intoxication, epilepsy, hypoglycemia, electrolyte abnormalities, infections, and psychiatric abnormalities.^2^ Among them, epilepsy is considered one of the most serious neurological disorders, affecting over 70 million people worldwide.^4^ Its incidence follows a bimodal distribution, with the highest risk observed in infants and the elderly. Furthermore, it has been pointed out that approximately 30% of elderly‐onset epilepsy cases experience status epilepticus at the time of initial onset, and among them, the frequency of NCSE is high.^5^ NCSE is defined as a condition characterized by nonconvulsive clinical neurological symptoms caused by EEG seizure activity lasting for more than 30 min.^6^ The symptoms of NCSE have been reported to manifest as altered consciousness in the form of confusion or coma, as well as language impairment, myoclonus‐like movements, anxiety, excitement, delirium, and extrapyramidal symptoms.^7^ In this case, the patient initially regained consciousness after completion of general anesthesia. However, shortly thereafter, he experienced loss of consciousness that persisted for more than 30 min. Although EEG could not be performed during the episode of impaired consciousness, it is likely that the patient experienced NCSE characterized by the predominant symptom of DOC. Nonconvulsive status epilepticus is a condition that is often observed in patients with unexplained DOC in whom no clear cause is identified by imaging. The diagnosis and, hence, treatment of this condition are often missed because it does not present with obvious seizures. However, if treatment is delayed, it can lead to irreversible brain damage. Therefore, early diagnosis and prompt treatment are crucial.^8^ The risk factors for the development of NCSE include prodromal generalized tonic–clonic seizures, a history of epilepsy, advanced age, female gender, and a history of brain injury. Therefore, in the presence of any of these risk factors in a patient with unexplained DOC, NCSE should be considered in the differential diagnosis.^9^, ^10^ In this patient, there were no specific risk factors except for advanced age, which made the diagnosis challenging.

The treatment of NCSE often involves the administration of benzodiazepines, which have been found to be effective.^11^, ^12^ In this case, a significant improvement in consciousness was observed after the administration of diazepam. Other medications, such as phenytoin and valproic acid, might also be considered in the treatment. Newer antiepileptic drugs, such as levetiracetam, which have fewer side effects and drug interactions, can also be used for the treatment of NCSE.^13^

It is indeed rare to observe the onset of NCSE immediately after the completion of general anesthesia for surgery. To our knowledge, there have been a few reported cases of NCSE occurring after neurosurgical procedures, but only one reported case of NCSE following non‐cranial surgery.^14^ Our experience suggests that when organic causes for postanesthetic impaired consciousness are ruled out, NCSE should be considered in the differential diagnosis. If feasible, immediate EEG monitoring should also be considered to aid in diagnosis.

In this case, although a definitive diagnosis of NCSE could not be made due to the unavailability of immediate EEG monitoring, improvement in consciousness level immediately following the administration of diazepam as a diagnostic and therapeutic intervention strongly suggested the possibility of NCSE. Postanesthetic DOC can often be attributed to residual effects of anesthetic drugs, making it challenging to diagnose NCSE. However, in elderly patients, NCSE should be considered in the differential diagnosis of DOC. Fortunately, in this case, the patient was managed without severe neurological complications.

## AUTHOR CONTRIBUTIONS

**Yumi Tsuzuki:** Conceptualization; writing – original draft. **Yusuke Ishida:** Conceptualization; writing – original draft; writing – review and editing. **Mikiko Tomino:** Writing – review and editing.

## FUNDING INFORMATION

None.

## CONFLICT OF INTEREST STATEMENT

The authors declare that they have no competing interests associated with this manuscript.

## ETHICS STATEMENT

Not applicable.

## CONSENT

Written informed consent was obtained from the patient for publication of this case report and accompanying images.

## Data Availability

The data that support the findings of this study are available from the corresponding author upon reasonable request.
